# Newcastle disease virus expressing clade 2.3.4.4b H5 hemagglutinin confers protection against lethal H5N1 highly pathogenic avian influenza in BALB/c mice

**DOI:** 10.3389/fvets.2025.1535274

**Published:** 2025-04-22

**Authors:** Deok-Hwan Kim, Seung-Hun Lee, Jiwon Kim, Jiho Lee, Ji-Hun Lee, Jei-hyun Jeong, Ji-yun Kim, Yang-Kyu Choi, Sungsu Youk, Chang-Seon Song

**Affiliations:** ^1^Avian Disease Laboratory, College of Veterinary Medicine, Konkuk University, Seoul, Republic of Korea; ^2^KHAV Co., Ltd., Seoul, Republic of Korea; ^3^Department of Microbiology, College of Medicine, Chungbuk National University, Cheongju, Republic of Korea; ^4^Southeast Poultry Research Laboratory, U.S. Department of Agriculture-Agricultural Research Service, U.S. National Poultry Research Center, Athens, GA, United States; ^5^Department of Laboratory Animal Medicine, College of Veterinary Medicine, Konkuk University, Seoul, Republic of Korea; ^6^Biomedical Research Institute, Chungbuk National University Hospital, Cheongju, Republic of Korea

**Keywords:** high pathogenic avian influenza, H5N1 clade 2.3.4.4b, Newcastle disease virus-vectored vaccine, mammalian infection and transmission, lung and brain viral load

## Abstract

The widespread H5 clade 2.3.4.4b highly pathogenic avian influenza virus (HPAI) poses a significant threat to both domestic and wild mammals because of its rapid genetic evolution, cross-species transmissibility, and host-range expansion. The increasing number of cases in mammalian species highlights the need for proactive measures driven by the One Health approach. In this study, we explored the potential use of previously developed a Newcastle disease virus (NDV)-vectored vaccine expressing clade 2.3.4.4b H5 hemagglutinin (rK148/22-H5) in a preclinical BALB/c mouse model. Two doses of intramuscular vaccination with viable (10^7^ EID_50_/0.1 mL) or inactivated (10^7^ EID_50_/0.1 mL) rK148/22-H5 provided protection against lethal H5N1 HPAI. A greater than 100-fold reduction in lung viral load was observed in the rK148/22-H5 vaccinated group compared to the control group. Consistently, co-housed contact mice in the vaccine group survived without evidence of infection, whereas those in the control group became infected and succumbed to the disease. The rK148/22-H5 vaccine demonstrated potential as a HPAI vaccine candidate for mammals, warranting further steps to advance this candidate vaccine into clinical trials in domestic and captive mammalian species.

## 1 Introduction

The highly pathogenic avian influenza (HPAI) H5N1 clade 2.3.4.4b, representing the fifth intercontinental wave of the Goose/Guangdong/96 (Gs/GD) lineage H5, emerged in Europe in 2020 (1). It has since become the most severe panzootic, affecting the largest geographical regions including Europe, North America, South America, Asia, Africa, Antarctica, and the Arctic (2–6). Unlike previous Gs/GD-lineage H5 waves, several wild and domestic mammal cases demonstrate cross-species transmissibility and host-range expansion of the current panzootic, indicating a shift in the patterns of mammalian infection (7). Whether through natural transmission or human-related exposure, a diverse range of wild and domestic mammals have been affected, including badgers, black bears, bobcats, coyotes, ferrets, fisher cats, foxes, leopards, opossums, pigs, raccoons, skunks, sea lions, and wild otters (8–11). Although most of these have been “dead-end” infections, attributed to direct contact from animals preying on and ingesting infected birds (12), three recent cases have shown potential mammal-to-mammal transmission: (1) New England seals in United States, (2) mink farms in Spain, and (3) seal lions in Peru (13–15). Moreover, dairy cows in the United States have been infected with HPAI H5N1 (16). Viable viruses have been detected in the raw milk of infected cows, leading to subsequent infections in cats that consume it (17). A suspected case of farm worker infection from an infected cow has been reported in Texas (18). These unprecedented situations highlight the imminent risk of the zoonotic spread of the current HPAI H5N1, thereby posing a threat to animal and public health.

Newcastle disease virus (NDV), a member of the genus Orthoavulavirus and the family *Paramyxoviridae*, is a non-segmented, negative-sense, single-stranded RNA virus. It is comprised of six structural proteins: nucleoprotein (NP), phosphoprotein (P), matrix protein (M), fusion protein (F), hemagglutinin-neuraminidase (HN), and large protein (L) (19). Since the late 1990s, NDV has been studied as vaccine platform by inserting foreign genes between NDV genes and expressing foreign proteins (20). Different lentogenic and mesogenic NDV strains have been studied as vaccine platform for influenza, severe acute respiratory syndrome coronavirus 2 (SARS-CoV-2), human immunodeficiency virus (HIV), ebolavirus (EBOV), Japanese encephalitis virus (JEV), and other pathogens (21). The fact that the NDV-vectored vaccine can replicate at high titers in both embryonated eggs and cell lines offers an economic advantage, as it allows the use of existing large-scale embryonated egg-based production systems (22). The NDV-vector vaccine platform has demonstrated a high level of safety in humans to the extent that clinical phase 1 and 2/3 trials for SARS-CoV-2 vaccine candidates, both live and inactivated, are being conducted (23). In addition, the ability of the surface HN glycoprotein to bind to sialic acid receptors, which are widely distributed on the cell surface across different species, highlights the potential of an NDV-based vector as a versatile vaccine platform applicable to other mammalian species, including cattle, sheep, pigs, dogs, cats, minks, and ferrets (24–29).

In the present study, we investigated the potential veterinary applications of the previously developed recombinant NDV-vectored HPAI H5 clade 2.3.4.4 b (rK148/22-H5) (30). As part of the preclinical trials, cellular and humoral immune responses to the live vaccine and the gel-adjuvanted inactivated vaccine were evaluated in BALB/c mice. Protection against lethal H5N1 HPAI was assessed by measuring survival rates, clinical symptoms, lung viral load, and transmission to unvaccinated contact mice.

## 2 Materials and methods

### 2.1 Viruses and cells

HPAI clade 2.3.4.4b H5N1 virus (K22-862, A/spot-billed duck/Korea/K22-862-1/2022; GISAID accession no. EPI ISL 15944665) was propagated in 10-d-old specific pathogen-free (SPF) embryonated chicken eggs (ECEs), and the 50% egg infective dose (EID_50_) of the virus was measured (31). All experiments involving viable HPAI H5N1 viruses were conducted at a Biosafety Level (BSL)-3 facilities (Konkuk University) in accordance with procedures approved by the Konkuk University Institutional Biosafety Committee (approval no. KUIBC-2023-14). Chicken fibroblasts [DF-1 cells, CRL-12203, American Type Culture Collection (ATCC), United States] were grown in Dulbecco’s modified Eagle’s medium (DMEM) supplemented with 8% fetal bovine serum (FBS; Biowest, Nuaillé, France) and antibiotics at 37°C in a 5% CO_2_ incubator.

### 2.2 Vaccine preparation

NDV-expressing H5 HA vaccine was prepared as described previously (30). Briefly, rK148/22-H5 was developed by inserting the HA gene of HPAIV H5N1 K22-862-1, isolated in November 2022, between the P and M genes of NDV K148/08 (*Anas platyrhynchos*/Korea/K148/2008; GenBank accession no. KF724899). The rescued rK148/22-H5 was propagated in 10-d-old SPF ECEs eggs at 37°C for 3 d, reaching a final titer of 10^9.5^ EID_50_/mL. The virus was diluted with phosphate-buffered saline (PBS) for vaccine preparation: the live vaccine was diluted to 10^8^ EID_50_/mL, and the inactivated vaccine was diluted to 10^8.5^ EID_50_/mL. For the gel-adjuvanted inactivated vaccine, one portion of the formalin-inactivated allantoic fluid of HPAI rK148/22-H5 was mixed with 10 portions of hydrogels (CTCVAC, Hongcheon, Korea), and then homogenized according to the instructions of manufacturer, resulting in a final vaccine concentration of 10^8^ EID_50_/mL.

### 2.3 Immunization and HPAIV infection

All vaccinations were performed at the BSL-2 facility, and infections were performed at the animal BSL-3 facility at Konkuk University. Animal immunization and infection studies were reviewed, approved, and supervised by the Institutional Animal Care and Use Committee of Konkuk University (approval no. KU23178).

Fifty-seven 6-week-old BALB/c mice (Orient Bio, Seong-Nam, South Korea) were divided into three groups (Table 1). Animal care and experimental procedures were conducted in compliance with the approved guidelines of the Institutional Animal Care and Use Committee. Each of the 16 mice in the groups received 0.1 mL of the prepared vaccines intramuscularly, administered twice at a 2-week interval, as follows: 10^7^ EID_50_ of the gel-adjuvanted inactivated rK148/22-H5 for group 1 (G1) and 10^7^ EID_50_ of the viable rK148/22-H5 for group 2 (G2). As a control, group 3 (G3) received 0.1 mL of PBS as a mock vaccination.

**Table 1:** Vaccine dose, vaccine type, and number of mice tested in the rK148/22-H5 vaccine experiment.

Group	Vaccine	Vaccine dose	Vaccine type	Age	Total number of mice vaccinated	The number of contact mice^a^
G1	rK148/22-H5	10^7.0^ EID_50_	Inactivated	6w	16	3
G2	rK148/22-H5	10^7.0^ EID_50_	Live		16	3
G3	PBS	–	–		16	3

a 2 dpc, mice were co-housed in the same cage for direct contact.

Blood serum samples were collected weekly from seven randomly selected mice in each group, starting for initiation of vaccination and continuing until the HPAIV challenge. Two weeks after the booster vaccination, the vaccinated mice were challenged intranasally with 50 μL of 10^7.3^ EID_50_/mL of the HPAI H5N1 virus (K22-862). Three BALB/c mice (unvaccinated mice) per group were co-housed for direct contact at 2 d post challenge (dpc). Further, at 3 and 6 dpc, three mice per group were euthanized to extract lungs, spleens and small intestines. On day 12 of direct contact, lung and brain samples were collected to confirm viral transmission. The survival rate and body weight of HPAIV-infected mice and contact mice were observed daily until 14 dpc.

### 2.4 Serological analysis

The hemagglutinin inhibition (HI) titer against the NDV vector virus (K148/08) and the HPAI H5 clade 2.3.4.4b virus (K22-862) for each sample (*n* = 84) was determined using a standard protocol (32). Specifically, serum was combined with a receptor-destroying enzyme (Denka Seiken, Japan) at a 1:3 dilution ratio, and the mixture was incubated at 37°C for 18 h, and subsequently inactivated at 56°C for 30 min. The inactivated serum was serially diluted two-fold with PBS in 96-well V-bottom plates. Four hemagglutination units of either K148/08 or HPAIV H5N1 (K22-862) were then added to each well and incubated for 40 min at 20–25°C. The incubated samples were mixed with equal volumes of 1% Turkey red blood cells in PBS. HI titers were reported as reciprocal log_2_ titers. The cutoff value for a positive HI response was defined as a titer of 2^2^ HIU or higher.

### 2.5 Splenocyte and interferon-gamma enzyme-linked immunospot assay

The splenocyte isolation and IFN-γ ELISpot assay were performed as described previously (33). In summary, mouse spleens were isolated and transferred to Roswell Park Memorial Institute (RPMI) 1,640 medium (Sigma-Aldrich, Irvine, United Kingdom) supplemented with 10% FBS (R10 RPMI 1640). Single cells were obtained by mincing the spleens through a 70 μm cell strainer (SPL Life Sciences, Korea) placed over a petri dish. The IFN-*γ* ELISpot assay was conducted using a 3,321-4APW-10 kit (Mabtech, Sweden), the HPAI H5 clade 2.3.4.4b (K22-862) antigen was heated at 80°C for 15 min before use. The quantification of spots was carried out using an AID iSpot system (AID Autoimmun Diagnostika GmbH, Germany).

### 2.6 Lung and brain viral load against HPAIV

Each lung and brain tissue were homogenized using a mortar and pestle, and the resulting material was preserved in 10% (w/v) PBS. After centrifugation at 3,000 × g for 10 min, the supernatant was collected. DF-1 cell monolayers were then inoculated with 100 μL of the supernatant (diluted 10-fold in DMEM). The inoculated cells were incubated at 37°C with 5% CO_2_ for 1 h, followed by overlaying with 100 μL of 4% FBS in DMEM. The cytopathic effect (CPE) was observed at 4 d post-inoculation, and the 50% tissue culture infectious dose (TCID_50_) was calculated.

### 2.7 Histopathological evaluation

The lungs harvested from euthanized mice were fixed with 10% neutral-buffered formalin, processed, and embedded in paraffin blocks for hematoxylin and eosin (H&E) staining. Histopathological lesions in the lungs were scored as previously described (34). The lungs were assessed for interstitial pneumonia, perivascular edema, and bronchiolitis. The severity of pneumonia and perivascular edema was scored on a scale of 0–5, whereas bronchiolitis was scored on a scale of 0–3. These scores were summed to obtain a total score, which represented the final histopathological assessment of the lung lesions.

### 2.8 Statistical analysis

The viral titer was determined as TCID_50_ using the Reed–Muench method (35). Statistical analyses were performed using GraphPad Prism 8.0 (Boston, MA, United States).^1^ Statistical significance was determined using a one-way analysis of variance (ANOVA) with Tukey’s correction test. A one-tailed *t*-test was used to compare the three groups. When the sample size was three, Friedman test was used for non-parametric statistical analysis, and Dunn’s test was used for multiple comparisons (IFN-*γ* ELISpot, lung and brain viral load, histopathological score). Statistical significance was set at *p* < 0.05.

## 3 Results

### 3.1 Immune response to the rK148/22-H5 vaccines

**Figure 1:**
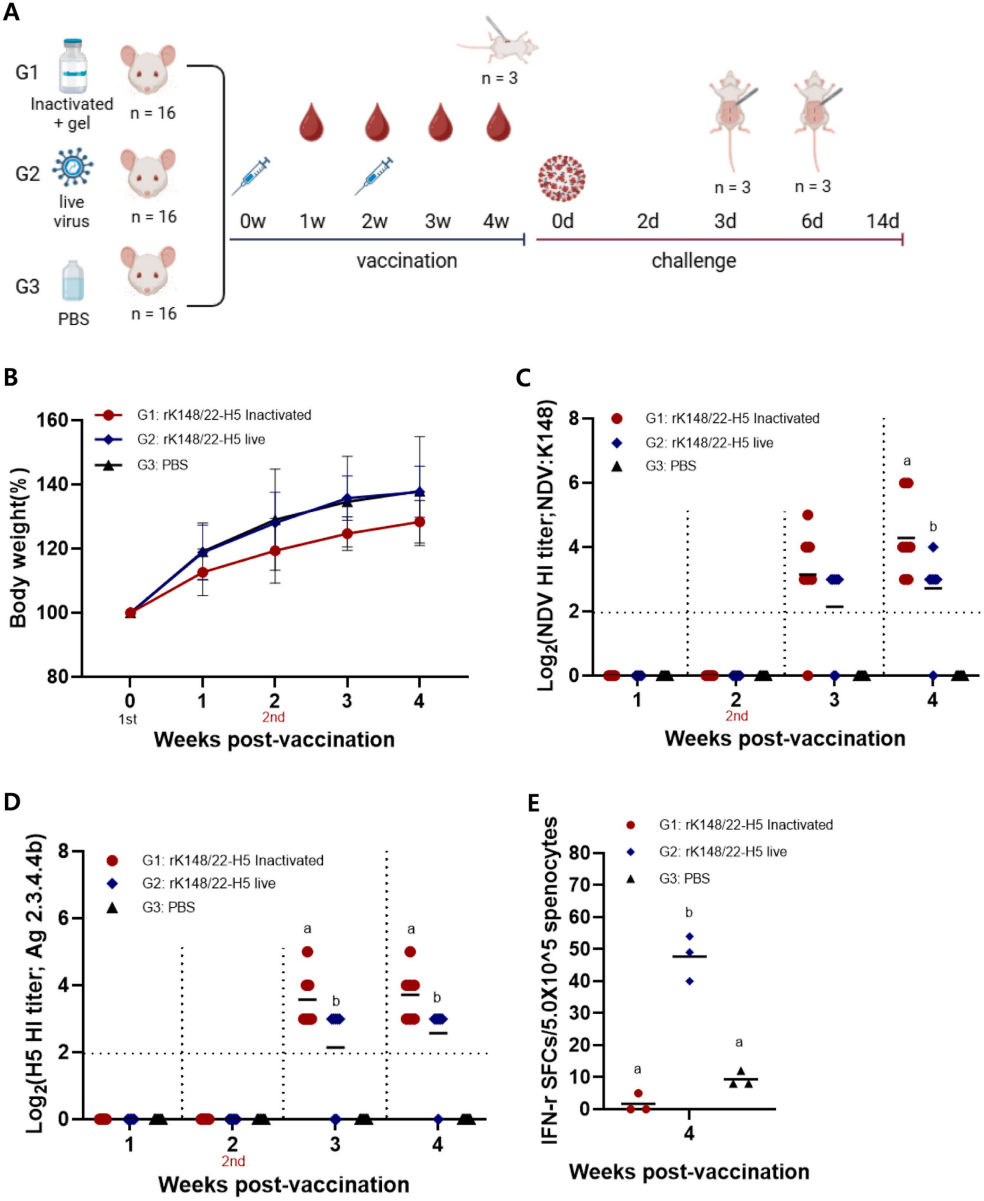
Immune response to live rK148/22-H5 and gel-adjuvanted inactivated rK148/22-H5 vaccines in BALB/c mice. **(A)** rK148/22-H5 intramuscular vaccination and challenge schedule. **(B)** Change in body weight measured weekly until 4 weeks post-vaccination (wpv). **(C,D)** HI assay for NDV or HPAI H5N1 in serum samples (*n* = 84) of vaccinated BALB/c mice. BALB/c mice with NDV and HPAIV HI titer <2 log_2_ were regarded as seronegative. **(E)** Splenocyte and IFN-*γ* ELISpot was performed using euthanized mice (*n* = 3 animals each) at 2 wpv with the secondary vaccine. HPAI H5N1 (K22-862) was used as the antigen.

Six-week-old BALB/c mice were intramuscularly vaccinated with either the gel-adjuvanted inactivated rK148/22-H5 (100 μL of 10^8.0^ EID_50_/mL) or the live rK148/22-H5 (100 μL of 10^8.0^ EID_50_/mL) (Figure 1A; Table 1). The humoral immune response was assessed using an HI assay. No significant differences were observed in body weight between the groups (Figure 1B). HI titers against both the NDV vector and H5 HA were detectable as early as 1 week after booster vaccination in both the inactivated and live vaccine groups. At the challenge time point, the mean HI titers against both NDV and H5 HA were significantly higher in the gel-adjuvanted inactivated rK148/22-H5 group (2^4.3^ and 2^3.7^ HAU, respectively) compared to the live rK148/22-H5 group (2^2.7^ and 2^2.6^ HAU, respectively) (Figures 1C,D). To assess the cellular immune response, an IFN-*γ* ELISpot was performed using splenocytes from three mice per each group, 2 weeks after the boost vaccination. The mean IFN-γ spots formed were significantly higher in the live rK148/H5 group (*n* = 47.7 ± 5.8), as compared to the control group (*n* = 9.3 ± 1.9) and the inactivated rK148/22-H5 group (*n* = 1.7 ± 2.4) (Figure 1E). These results indicated that rK148/22-H5 successfully induced a humoral immune response against the target antigen in both live and inactivated forms. However, only the live form induced a cellular immune response.

### 3.2 Survival rate, symptoms, lung viral load, and contact transmission

**Figure 2:**
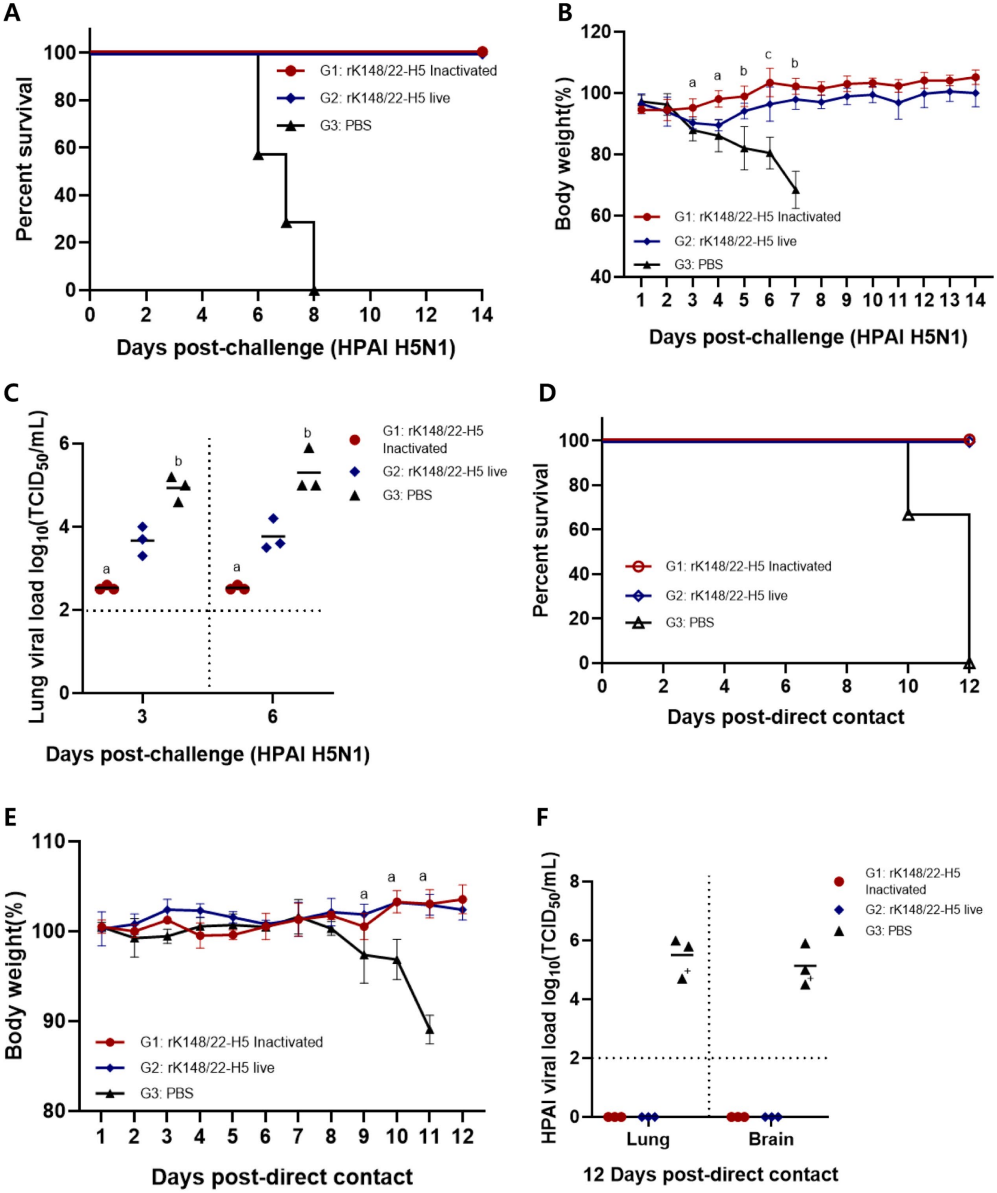
Survival rate and viral load after challenge with HPAIV H5N1 and direct contact. **(A,B)** Body weight changes and survival rate at 14 dpc with HPAI H5N1 (K22-862). For the challenge, 100 μL each of HPAI H5N1 (10^7.0^ EID_50_/mL) was inoculated intranasally (*n* = 13). The “a” indicates the comparison of G1 with G2 and G3, “b” indicates the comparison of G1 and G2 with G3, and “c” indicates that all groups show statistical significance, respectively (*p* > 0.05) **(C)** After challenge with HPAI H5N1, mice were euthanized at 3 and 6 dpc (*n* = 3 animals each) to measure the viral load in the lungs. BALB/c mice with viral load < 10^2^ TCID_50_/mL were regarded as negative. Groups with at least one shared superscript letter indicate that no significant statistical differences between pairwise comparisons within the same day were observed (*p* > 0.05). **(D,E)** Body weight changes and survival rate at 12 d direct-contact with HPAI H5N1 (K22-862) challenged mice (*n* = 3). **(F)** After direct-contact with HPAI H5N1 challenged mice, mice were euthanized at 12 d direct-contact (*n* = 3 animals each) to measure the viral load in the lungs and brains. BALB/c mice with viral load < 10^2^ TCID_50_/mL were regarded as negative (“+” died at 10 d direct-contact in group 3).

Two weeks after the booster vaccination, BALB/c mice were challenged with HPAI H5N1 (K22-862) (Figure 1A). All vaccinated mice survived for up to 14 dpc, whereas all control mice died 6–8 dpc (Figure 2A). The inactivated rK148/22-H5 group exhibited no significant changes in body weight until 14 dpc. The live rK148/22-H5 group showed significant weight loss compared to the inactivated vaccinated group at 3, 4, and 5 dpc; however, no significant weight difference was observed from day 6 onward. The PBS group exhibited a sharp and significant decrease in body weight at 3 dpc (Figure 2B). Among the contact mice in the PBS control group, two exhibited tremors due to neurological symptoms on day 11 and died on day 12 post-contact (Supplemental Video 1).

At 3 and 6 dpc, three mice from each group were euthanized to measure the lung viral load of the challenge virus. The inactivated rK148/22-H5 group showed lung viral load of 10^2.5^ TCID_50_/mL at 3 dpc and 6 dpc. The live rK148/22-H5 group showed lung viral load of 10^3.7^ TCID_50_/mL at 3 dpc and 10^3.8^ TCID_50_/mL at 6 dpc. The control group showed lung viral load of 10^4.9^ TCID_50_/mL at 3 dpc and 10^5.3^ TCID_50_/mL at 6 dpc, respectively. A significantly lower lung viral load was observed in the inactivated vaccine group compared to the control group. (Figure 2C).

To observe the extent of HPAIV transmission following vaccination, three mice from each group were cohoused via direct contact at 2 dpc (Figure 1A). All mice in contact with vaccinated mice survived with no clinical symptoms or body weight loss until 12 d after contact. In contrast, all contact mice co-housed with the control group died 10–12 d after contact, with a significant sharp decrease in body weight at 9 d after contact (Figures 2D,E). The contact mice that died showed high lung and brain viral load of 10^5.5^ TCID_50_/mL and 10^5.1^ TCID_50_/mL, respectively, whereas the contact mice co-housed with vaccinated mice showed no detectable viral titer (Figure 2F).

### 3.3 Histopathological analysis

**Figure 3:**
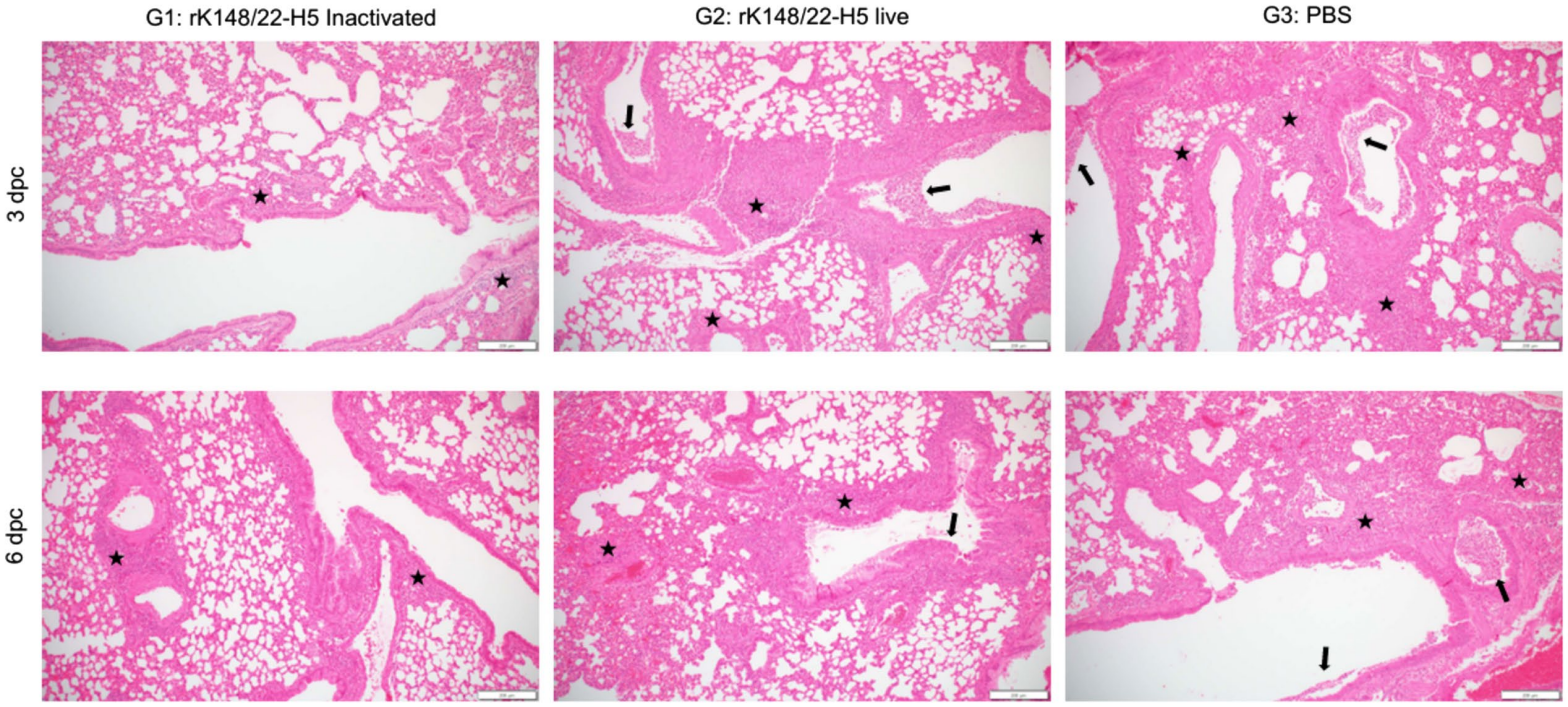
Histopathological analysis of lung after challenge with HPAIV H5N1. Representative H&E images of the lungs at 3 (upper panel) and 6 (lower panel) dpc after challenge with HPAI H5N1. The lungs were examined for interstitial pneumonia, perivascular edema, and bronchiolitis. Inflammatory cells infiltrating the interstitial space (black stars) and bronchiolar lumen (black arrows) are indicated. Scale bars, 200 μm.

The histopathological impact of HPAIV infection at 3 and 6 dpc was assessed in the lungs of BALB/c mice, as these are known to be affected by HPAIV in this model (Figure 3) (36). The lungs of infected mice exhibited typical features of interstitial pneumonia, characterized by the infiltration of inflammatory cells in the alveolar walls. The severity of pneumonia was the least pronounced in the inactivated rK148/22-H5 group at 3 and 6 dpc. Signs of perivascular edema, indicated by enlargement of the perivascular space with inflammatory cells, were not evident in any group. However, bronchiolitis, marked by inflammatory infiltrates within the bronchiolar lumen was prominent in the live rK148/22-H5 and control groups, whereas the mildest lesions were exhibited in the inactivated rK148/22-H5 group.

**Figure 4:**
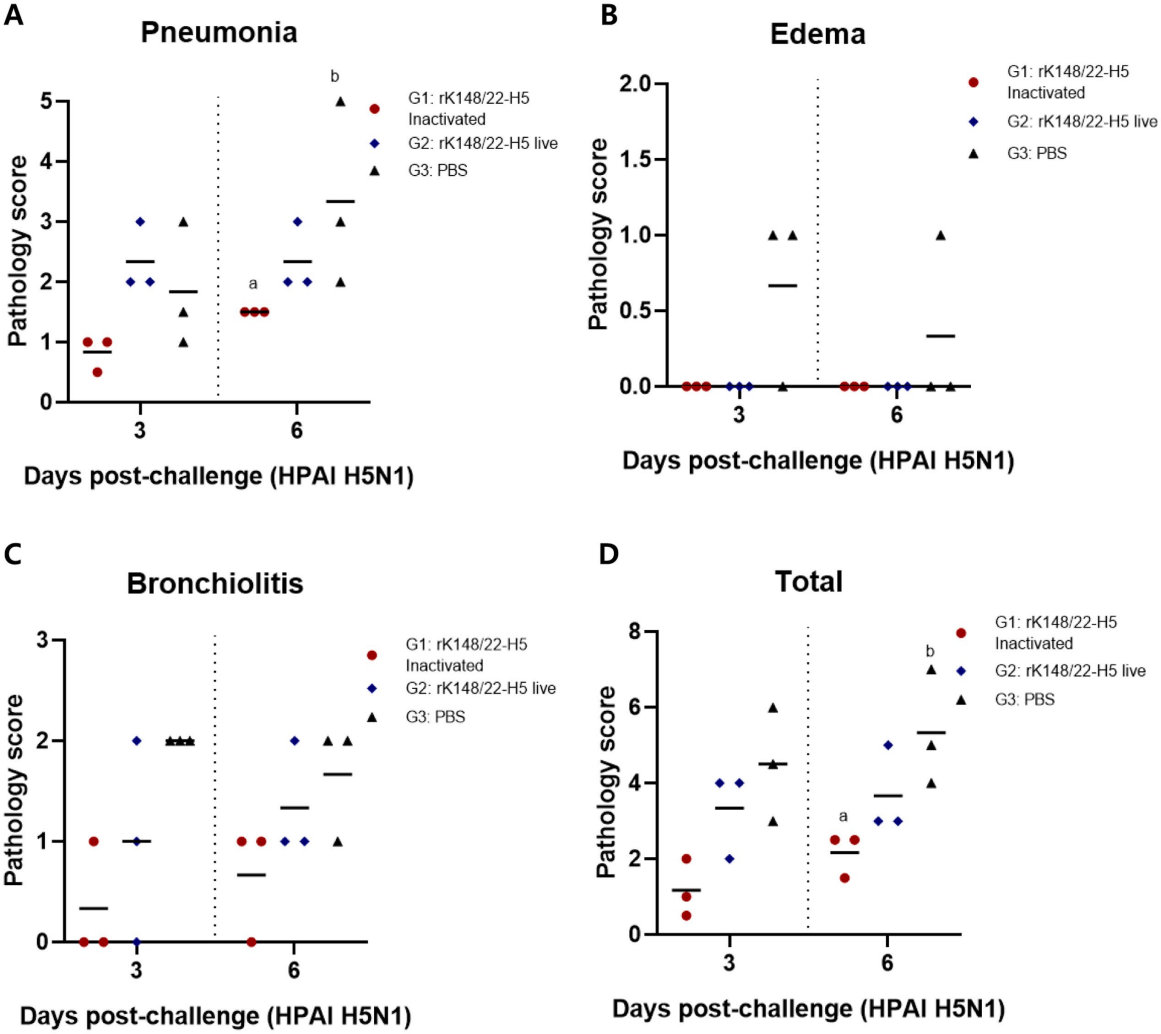
Histopathological scoring of lung after challenge with HPAIV H5N1. **(A)** Pneumonia score in the lungs after challenge. **(B)** Edema score after challenge in the lungs. **(C)** Bronchiolitis score after challenge in the lungs. **(D)** Total lungs score after challenge with HPAI H5N1 3 and 6 dpc. Graphs of groups with at least one shared superscript letter indicate that no statistically significant differences were observed between pairwise comparisons within the same day (*p* > 0.05).

Histopathological scoring of interstitial pneumonia revealed that the inactivated rK148/22-H5 group had the lowest pneumonia scores at 3 and 6 dpc compared to those of the other two groups (Figure 4A). Notably, at 6 dpc, the severity of pneumonia in the inactivated rK148/22-H5 group was significantly lower than that in the control group. The two vaccinated groups (G1 and G2) showed no signs of perivascular edema, whereas the control group displayed minimal edema (Figure 4B). The control group also exhibited higher levels of bronchiolitis than the other two groups (Figure 4C). This difference was particularly evident when compared to the inactivated rK148/22-H5 group, although the difference was not statistically significant. Overall, the total pathological scores demonstrated that the two vaccinated groups (G1 and G2) experienced lower levels of pathological changes in the lungs than the control group, with a significant difference between the control and inactivated rK148/22-H5 groups (Figure 4D).

## 4 Discussion

The HPAI H5N1 clade 2.3.4.4b that emerged in wild birds in 2020 has affected a broader range of mammalian species compared to earlier HPAI outbreaks. From 2003 to 2019, 12 species of mammals were infected with HPAIVs; however, from 2020 to 2023, 48 mammalian species were infected, including terrestrial and marine wildlife (7, 37). This expanding host range poses a growing threat to domestic animals. The clade 2.3.4.4b H5N1 initially emerged in milk cows in the United States in March 2024 and was subsequently transmitted to farm workers (18). Previous studies have shown that the HPAI H5N1 clade 2.3.4.4b readily acquires mutations that enable adaptation to mammals (38–40), and such mutations have been demonstrated in infected wildlife mammals (37). Therefore, vaccination should be prepared in advance as a potential option for controlling H5N1 if the virus escalates to a panzootic state (41).

Vaccination with rK148/22-H5 induced antibody responses against HPAI H5N1 in both live and gel-adjuvanted inactivated forms. In a previous study, western blotting confirmed the expression of H5 HA in purified rK148/22-H5 (33). Thus, the HA protein delivered on the surface of the rK148/22-H5 virion likely mediated a humoral response against H5 HA (42). An additional advantage of the inactivated rK148/22-H5 vaccine is its potential as an alternative when live recombinant NDV vaccines are unsuitable for use in mammalian species owing to regulatory restrictions and environmental exposure concerns. In the mice administered the live rK148/22-H5 vaccine, the cellular immune response was confirmed by the production of IFN-*γ* from spleen cells in the live rK148/22-H5 vaccine group. Because the NDV receptor can bind to surface glycoproteins containing ubiquitous sialic acid residues (43), it was hypothesized that rK148/22-H5 enters cells at the injection site, undergoes limited rounds of replication, and is subsequently cleared by the immune response. This limited replication likely stimulates both cellular and humoral immune responses by targeting the inserted H5 HA. Although antibody titers were lower than those induced by the inactivated vaccine, cellular immunity can offer advantage of broader protection against antigenically distinct H5 viruses (44). Upon lethal HPAI H5N1 infection, both inactivated and live rK148/22-H5 vaccines provided complete protection, with no transmission observed in mice in direct contact. The vaccinated groups showed a significant reduction in lung viral load and attenuation of lung lesions, with the inactivated vaccine having a greater impact than the controls. This suggest that the rK148/22-H5 vaccine has the potential to mitigate typical respiratory symptoms, such as pneumonia, edema, and bronchiolitis, which commonly impair lung function and lead to clinical symptoms such as dyspnea and cough.

Previous studies on rK148/22-H5 as a poultry vaccine have demonstrated its efficacy in providing protection against NDV as well as against HPAI in poultry (30). In this study, we extended the applicability of rK148/22-H5 to mammalian species and investigated its potential utility as a single strain for both avian and mammalian species. The NDV-vectored HPAI H5 vaccine can be grown to high titers in embryonated eggs, similar to influenza, which allows the use of existing influenza vaccine production facilities for its manufacturing (22). The production of high-titer viruses can significantly contribute to vaccine costs reduction. The K148/08 strain, used as the backbone of the NDV vector in this study, exhibits high thermal stability (45), which facilitates transport to target destinations after production, making it suitable for widespread global use. The rK148/22-H5 can be used in both live and inactivated oil-emulsified forms, making it adaptable to various vaccine demands. Therefore, a single strain can be used to produce both live and inactivated vaccines. It can also be used in prime-live and boost-inactivated strategies to maximize humoral and cellular immune responses (46). The influenza virus is one of the most rapidly evolving viruses, and the transmission of newly evolved strains occurs rapidly (47). Although extensive research has been conducted to develop a universal vaccine as an optimal approach to address rapidly mutating RNA viruses, the influenza virus exhibits significant subtype diversity; thus, no universal vaccine has yet been commercialized (48). As an alternative approach to combat influenza, a vaccine platform that can keep pace with its rapid evolution is necessary. When developed as a platform, the NDV vector vaccine allows the insertion of foreign genes, enabling vaccine production within a few months (49).

Several factors must be considered to apply rK148/22-H5 in mammalian models. Because of the broad range of mammalian species, it is crucial to prioritize specific target groups. First, animals housed in zoological settings can be considered. A zoo is a facility in which various wildlife species are housed, ranging from birds, which are the primary targets of HPAI, to diverse mammalian species. In 2016, HPAI outbreaks in zoo-housed birds in Japan resulted in high mortality. Phylogenetic analysis indicates direct viral transmission among birds within each zoo (50). Cases of felid species such as tigers succumbing to reassorted HPAI infections within zoos have also been reported (51, 52). Since 2020, HPAI H5N1 clade 2.3.4.4b has demonstrated significantly increased infectivity and mortality rates in marine mammals compared to previous HPAIV strains (53). When an outbreak of HPAI occurs in a zoo, there is a high potential for transmission to other animals within the zoo and an increased risk of spreading to visitors. Second, the control measures should be considered for livestock populations. Cattle and pigs are representative of the livestock industry. In April 2024, the first case of HPAI was reported in cattle in the United States with documented cases of HPAI transmission to cats and humans (54). Although no cases of mass infection with HPAIV in pigs have been reported to date, pigs possess both avian and mammalian sialic acid receptors. This suggests the potential for the emergence of HPAIV adapted to mammalian sialic acid receptors in the event of mixed infections (55). Third, the use of vaccines in companion animals should be considered. The primary companion animals of mammals are dogs and cats. The case of mass infection in cats in Poland in July 2023 suggests that the HPAI H5N1 clade 2.3.4.4b, which emerged in 2020, has evolved to be more adapted to cats (56). In April 2023, cases of asymptomatic HPAI were reported in cats and dogs maintained on poultry farms in Italy (8). Asymptomatic infections in companion animals pose a risk as they may facilitate transmission among animals and potentially spread the virus to their owners, warranting caution. In the present study, mice weighing approximately 15–20 g were used to evaluate vaccine efficacy. However, the mammals mentioned above range in weight from at least 1 kg to as much as 400 kg, creating a significant discrepancy compared with the dosage used in mice. Previous studies evaluating the efficacy of NDV vector vaccines in mammals have shown that they can be applied in a range of species, including dogs, cats, pigs, and horses. A dose of 10^7.3^ EID_50_ has been shown to provide sufficient vaccine efficacy (28). In large animals such as pigs and horses, a dose of 10^9.0^ EID_50_ was required to achieve sufficient vaccine efficacy (27, 57). The rK148/22-H5 can proliferate up to 10^9.5^ EID_50_/mL in embryonated eggs, making it suitable as a vaccine for large animals without the need for further concentration. Wildlife vaccination is necessary to reduce the risk of extinction and zoonotic transmission; however, vaccines that provide complete immunity are rare, and imperfect vaccination may pose epidemiological, ecological, and evolutionary challenges. Disease prevention vaccines may weaken herd immunity and increase virulence. Therefore, approaches such as trait-based vaccination, modeling tools, and ecosystem-level vaccine safety assessments should be considered to address these issues and carefully evaluate vaccine use (58, 59).

In this study, intramuscular administration of live or gel-adjuvanted inactivated rK148/22-H5 resulted in a 100% survival rate and significantly reduced lung viral load after HPAIV infection. Moreover, vaccination effectively prevented transmission to mice in direct contact within the same cage. rK148/22-H5 is a promising vaccine candidate that provides protection against the HPAIV H5N1 clade 2.3.4.4b in mammals.

## Data Availability

The datasets presented in this study can be found in online repositories. The names of the repository/repositories and accession number(s) can be found in the article/Supplementary material.

## Footnotes

^1^ www.graphpad.com

## Supplementary material

Supplemental Video 1 Among the contact mice in the PBS control group, two exhibited tremors due to neurological symptoms on day 11.

## References

[1] Lewis NS Banyard AC Whittard E Karibayev T Al Kafagi T Chvala I. Emergence and spread of novel H5n8, H5n5 and H5n1 clade 2.3.4.4 highly pathogenic avian influenza in 2020. Emerg Microbes Infect. (2021) 10:148–51. doi: 10.1080/22221751.2021.1872355, PMID: 33400615 PMC7832535

[2] Cho AY Si Y-J Lee D-Y Kim D-J Kim D Jeong H. Index case of H5n1 clade 2.3. 4.4 B highly pathogenic avian influenza virus in wild birds, South Korea, November 2023. Front Vet Sci. (2024) 11:1366082. doi: 10.3389/fvets.2024.1366082, PMID: 38699674 PMC11064161

[3] Komu JG Nguyen HD Takeda Y Fukumoto S Imai K Takemae H. Challenges for precise subtyping and sequencing of a H5n1 clade 2.3.4.4b highly pathogenic avian influenza virus isolated in Japan in the 2022-2023 season using classical serological and molecular methods. Viruses. (2023) 15:2274. doi: 10.3390/v15112274, PMID: 38005950 PMC10675786

[4] Fusaro A Gonzales JL Kuiken T Mirinavičiūtė G Niqueux É Ståhl K. Avian influenza overview December 2023-march 2024. EFSA J. (2024) 22:e8754. doi: 10.2903/j.efsa.2024.8754, PMID: 38550271 PMC10977096

[5] Banyard AC Bennison A Byrne AMP Reid SM Lynton-Jenkins JG Mollett B. Detection and spread of high pathogenicity avian influenza virus H5n1 in the Antarctic region. Nat Commun. (2024) 15:7433. doi: 10.1038/s41467-024-51490-8, PMID: 39227574 PMC11372179

[6] Gass JD Jr Dusek RJ Hall JS Hallgrimsson GT Halldórsson HP Vignisson SR. Global dissemination of influenza a virus is driven by wild bird migration through Arctic and subarctic zones. Mol Ecol. (2023) 32:198–213. doi: 10.1111/mec.16738, PMID: 36239465 PMC9797457

[7] Plaza PI Gamarra-Toledo V Euguí JR Lambertucci SA. Recent changes in patterns of mammal infection with highly pathogenic avian influenza a(H5n1) virus worldwide. Emerg Infect Dis. (2024) 30:444–52. doi: 10.3201/eid3003.231098, PMID: 38407173 PMC10902543

[8] Moreno A Bonfante F Bortolami A Cassaniti I Caruana A Cottini V. Asymptomatic infection with clade 2.3.4.4b highly pathogenic avian influenza a(H5n1) in carnivore pets, Italy, April 2023. Euro Surveill. (2023) 28:2300441. doi: 10.2807/1560-7917.Es.2023.28.35.2300441 37650905 PMC10472752

[9] Rimondi A Vanstreels RET Olivera V Donini A Lauriente MM Uhart MM. Highly pathogenic avian influenza a(H5n1) viruses from multispecies outbreak, Argentina, august 2023. Emerg Infect Dis. (2024) 30:812–4. doi: 10.3201/eid3004.231725, PMID: 38413243 PMC10977829

[10] Vreman S Kik M Germeraad E Heutink R Harders F Spierenburg M. Zoonotic mutation of highly pathogenic avian influenza H5n1 virus identified in the brain of multiple wild carnivore species. Pathogens. (2023) 12:168. doi: 10.3390/pathogens12020168, PMID: 36839440 PMC9961074

[11] Elsmo EJ Wünschmann A Beckmen KB Broughton-Neiswanger LE Buckles EL Ellis J. Highly pathogenic avian influenza a(H5n1) virus clade 2.3.4.4b infections in wild terrestrial mammals, United States, 2022. Emerg Infect Dis. (2023) 29:2451–60. doi: 10.3201/eid2912.230464, PMID: 37987580 PMC10683806

[12] Gilbertson B Subbarao K. Mammalian infections with highly pathogenic avian influenza viruses renew concerns of pandemic potential. J Exp Med. (2023) 220:e20230447. doi: 10.1084/jem.20230447, PMID: 37326966 PMC10276204

[13] Puryear W Sawatzki K Hill N Foss A Stone JJ Doughty L. Highly pathogenic avian influenza a(H5n1) virus outbreak in New England seals, United States. Emerg Infect Dis. (2023) 29:786–91. doi: 10.3201/eid2904.221538, PMID: 36958010 PMC10045683

[14] Agüero M Monne I Sánchez A Zecchin B Fusaro A Ruano MJ. Highly pathogenic avian influenza a(H5n1) virus infection in farmed minks, Spain, October 2022. Euro Surveill. (2023) 28:2300001. doi: 10.2807/1560-7917.Es.2023.28.3.2300001 36695488 PMC9853945

[15] Leguia M Garcia-Glaessner A Muñoz-Saavedra B Juarez D Barrera P Calvo-Mac C. Highly pathogenic avian influenza a (H5n1) in marine mammals and seabirds in Peru. Nat Commun. (2023) 14:5489. doi: 10.1038/s41467-023-41182-0, PMID: 37679333 PMC10484921

[16] Eisfeld AJ Biswas A Guan L Gu C Maemura T Trifkovic S. Pathogenicity and transmissibility of bovine H5n1 influenza virus. Nature. (2024) 633:426–32. doi: 10.1038/s41586-024-07766-6, PMID: 38977017 PMC11390473

[17] Burrough ER Magstadt DR Petersen B Timmermans SJ Gauger PC Zhang J. Highly pathogenic avian influenza a(H5n1) clade 2.3.4.4b virus infection in domestic dairy cattle and cats, United States, 2024. Emerg Infect Dis. (2024) 30:1335–43. doi: 10.3201/eid3007.240508, PMID: 38683888 PMC11210653

[18] Uyeki TM Milton S Abdul Hamid C Reinoso Webb C Presley SM Shetty V. Highly pathogenic avian influenza a(H5n1) virus infection in a dairy farm worker. N Engl J Med. (2024) 390:2028–9. doi: 10.1056/NEJMc2405371, PMID: 38700506

[19] Alexander DJ. Newcastle disease and other avian paramyxoviruses. Rev Sci Tech. (2000) 19:443–62. doi: 10.20506/rst.19.2.1231, PMID: 10935273

[20] Nakaya T Cros J Park MS Nakaya Y Zheng H Sagrera A. Recombinant Newcastle disease virus as a vaccine vector. J Virol. (2001) 75:11868–73. doi: 10.1128/jvi.75.23.11868-11873.2001, PMID: 11689668 PMC114773

[21] Fulber JPC Kamen AA. Development and scalable production of Newcastle disease virus-vectored vaccines for human and veterinary use. Viruses. (2022) 14:975. doi: 10.3390/v14050975, PMID: 35632717 PMC9143368

[22] Sparrow E Wood JG Chadwick C Newall AT Torvaldsen S Moen A. Global production capacity of seasonal and pandemic influenza vaccines in 2019. Vaccine. (2021) 39:512–20. doi: 10.1016/j.vaccine.2020.12.018, PMID: 33341308 PMC7814984

[23] Ponce-de-León S Torres M Soto-Ramírez LE Calva JJ Santillán-Doherty P Carranza-Salazar DE. Interim safety and immunogenicity results from an Ndv-based Covid-19 vaccine phase I trial in Mexico. NPJ Vacc. (2023) 8:67. doi: 10.1038/s41541-023-00662-6, PMID: 37164959 PMC10170424

[24] Khattar SK Collins PL Samal SK. Immunization of cattle with recombinant Newcastle disease virus expressing bovine Herpesvirus-1 (Bhv-1) glycoprotein D induces mucosal and serum antibody responses and provides partial protection against Bhv-1. Vaccine. (2010) 28:3159–70. doi: 10.1016/j.vaccine.2010.02.051, PMID: 20189484 PMC3428038

[25] Kortekaas J de Boer SM Kant J Vloet RP Antonis AF Moormann RJ. Rift Valley fever virus immunity provided by a paramyxovirus vaccine vector. Vaccine. (2010) 28:4394–401. Epub 20100429. doi: 10.1016/j.vaccine.2010.04.048, PMID: 20434545

[26] Kumar R Kumar V Kekungu P Barman NN Kumar S. Evaluation of surface glycoproteins of classical swine fever virus as immunogens and reagents for serological diagnosis of infections in pigs: a recombinant Newcastle disease virus approach. Arch Virol. (2019) 164:3007–17. doi: 10.1007/s00705-019-04425-4, PMID: 31598846

[27] Kong D Wen Z Su H Ge J Chen W Wang X. Newcastle disease virus-vectored Nipah encephalitis vaccines induce B and T cell responses in mice and Long-lasting neutralizing antibodies in pigs. Virology. (2012) 432:327–35. doi: 10.1016/j.virol.2012.06.001, PMID: 22726244

[28] Ge J Wang X Tao L Wen Z Feng N Yang S. Newcastle disease virus-vectored rabies vaccine is safe, highly immunogenic, and provides Long-lasting protection in dogs and cats. J Virol. (2011) 85:8241–52. doi: 10.1128/jvi.00519-11, PMID: 21632762 PMC3147977

[29] Ge J Wang X Tian M Gao Y Wen Z Yu G. Recombinant Newcastle disease viral vector expressing hemagglutinin or fusion of canine distemper virus is safe and immunogenic in minks. Vaccine. (2015) 33:2457–62. doi: 10.1016/j.vaccine.2015.03.091, PMID: 25865465

[30] Kim DH Lee SH Kim J Lee J Jeong JH Kim JY. Efficacy of live and inactivated recombinant Newcastle disease virus vaccines expressing clade 2.3.4.4b H5 hemagglutinin against H5n1 highly pathogenic avian influenza in Spf chickens, broilers, and domestic ducks. Vaccine. (2024) 42:3756–67. doi: 10.1016/j.vaccine.2024.04.088, PMID: 38724417

[31] Spackman E Killian ML. Avian influenza virus isolation, propagation, and titration in Embryonated chicken eggs. Methods Mol Biol. (2020) 2123:149–64. doi: 10.1007/978-1-0716-0346-8_12, PMID: 32170687

[32] OIE. Terrestrial manual chapter 3.3.4: avian influenza. OIE (2021).

[33] Kim DH Lee J Youk S Jeong JH Lee DY Ju HS. Intramuscular administration of recombinant newcastle disease virus expressing SARS-CoV-2 spike protein protects Hace-2 Tg mice against Sars-Cov-2 infection. Vaccine. (2023) 41:4787–97. doi: 10.1016/j.vaccine.2023.05.071, PMID: 37355454 PMC10266497

[34] Seo SM Son JH Lee JH Kim NW Yoo ES Kang AR. Development of transgenic models susceptible and resistant to sars-Cov-2 infection in FVB background mice. PLoS One. (2022) 17:e0272019. doi: 10.1371/journal.pone.0272019, PMID: 35881617 PMC9321403

[35] Reed LJ Muench H. A simple method of estimating fifty per cent Endpoints12. Am J Epidemiol. (1938) 27:493–7. doi: 10.1093/oxfordjournals.aje.a118408

[36] Cui P Shi J Wang C Zhang Y Xing X Kong H. Global dissemination of H5n1 influenza viruses bearing the clade 2.3.4.4b ha gene and Biologic analysis of the ones detected in China. Emerg Microbes Infect. (2022) 11:1693–704. doi: 10.1080/22221751.2022.2088407, PMID: 35699072 PMC9246030

[37] Peacock T Moncla L Dudas G VanInsberghe D Sukhova K Lloyd-Smith JO. The global H5n1 influenza Panzootic in mammals. Nature. (2024) 637:304–13. doi: 10.1038/s41586-024-08054-z, PMID: 39317240

[38] Jang SG Kim YI Casel MAB Choi JH Gil JR Rollon R. Ha N193d substitution in the Hpai H5n1 virus alters receptor binding affinity and enhances virulence in mammalian hosts. Emerg Microbes Infect. (2024) 13:2302854. doi: 10.1080/22221751.2024.2302854, PMID: 38189114 PMC10840603

[39] Charostad J Rezaei Zadeh Rukerd M Mahmoudvand S Bashash D Hashemi SMA Nakhaie M. A comprehensive review of highly pathogenic avian influenza (Hpai) H5n1: an imminent threat at doorstep. Travel Med Infect Dis. (2023) 55:102638. doi: 10.1016/j.tmaid.2023.102638, PMID: 37652253

[40] Mashaal D Mahmoud SH Müller C Abo Shama NM Kamer AA Abdelaziz AA. Differential impact of specific amino acid residues on the characteristics of avian influenza viruses in mammalian systems. Pathogens. (2022) 11:1385. doi: 10.3390/pathogens11111385, PMID: 36422635 PMC9698692

[41] Nohynek H Helve OM. One health, many interpretations: vaccinating risk groups against H5 avian influenza in Finland. Euro Surveill. (2024) 29:2400383. doi: 10.2807/1560-7917.Es.2024.29.25.2400383, PMID: 38904113 PMC11191420

[42] Veits J Wiesner D Fuchs W Hoffmann B Granzow H Starick E. Newcastle disease virus expressing H5 hemagglutinin gene protects chickens against Newcastle disease and avian influenza. Proc Natl Acad Sci USA. (2006) 103:8197–202. doi: 10.1073/pnas.0602461103, PMID: 16717197 PMC1472452

[43] Ferreira L Villar E Muñoz-Barroso I. Gangliosides and N-glycoproteins function as Newcastle disease virus receptors. Int J Biochem Cell Biol. (2004) 36:2344–56. doi: 10.1016/j.biocel.2004.05.011, PMID: 15313478

[44] Ge J Deng G Wen Z Tian G Wang Y Shi J. Newcastle disease virus-based live attenuated vaccine completely protects chickens and mice from lethal challenge of homologous and heterologous H5n1 avian influenza viruses. J Virol. (2007) 81:150–8. doi: 10.1128/jvi.01514-06, PMID: 17050610 PMC1797253

[45] Jeong S-H Lee D-H Kim B-Y Choi S-W Lee J-B Park S-Y. Immunization with a thermostable Newcastle disease virus K148/08 strain originated from wild mallard duck confers protection against lethal Viscerotropic Velogenic Newcastle disease virus infection in chickens. PLoS One. (2013) 8:e83161. doi: 10.1371/journal.pone.0083161, PMID: 24358260 PMC3865309

[46] Kardani K Bolhassani A Shahbazi S. Prime-boost vaccine strategy against viral infections: mechanisms and benefits. Vaccine. (2016) 34:413–23. doi: 10.1016/j.vaccine.2015.11.062, PMID: 26691569

[47] Kandeil A Patton C Jones JC Jeevan T Harrington WN Trifkovic S. Rapid evolution of a(H5n1) influenza viruses after intercontinental spread to North America. Nat Commun. (2023) 14:3082. doi: 10.1038/s41467-023-38415-7, PMID: 37248261 PMC10227026

[48] Madsen A Cox RJ. Prospects and challenges in the development of universal influenza vaccines. Vaccines. (2020) 8:361. doi: 10.3390/vaccines8030361, PMID: 32640619 PMC7563311

[49] de Swart RL Belov GA. Advantages and challenges of Newcastle disease virus as a vector for respiratory mucosal vaccines. Curr Opin Virol. (2023) 62:101348. doi: 10.1016/j.coviro.2023.101348, PMID: 37591130 PMC13262185

[50] Usui T Soda K Sumi K Ozaki H Tomioka Y Ito H. Outbreaks of highly pathogenic avian influenza in zoo birds caused by ha clade 2.3.4.4 H5n6 subtype viruses in Japan in winter 2016. Transbound Emerg Dis. (2020) 67:686–97. doi: 10.1111/tbed.13386, PMID: 31605424

[51] Hu T Zhao H Zhang Y Zhang W Kong Q Zhang Z. Fatal influenza a (H5n1) virus infection in zoo-housed tigers in Yunnan Province, China. Sci Rep. (2016) 6:25845. doi: 10.1038/srep25845, PMID: 27162026 PMC4861906

[52] He S Shi J Qi X Huang G Chen H Lu C. Lethal infection by a novel Reassortant H5n1 avian influenza a virus in a zoo-housed Tiger. Microbes Infect. (2015) 17:54–61. doi: 10.1016/j.micinf.2014.10.004 25461468

[53] Plaza PI Gamarra-Toledo V Rodríguez Euguí J Rosciano N Lambertucci SA. Pacific and Atlantic Sea lion mortality caused by highly pathogenic avian influenza a(H5n1) in South America. Travel Med Infect Dis. (2024) 59:102712. doi: 10.1016/j.tmaid.2024.102712, PMID: 38461878

[54] Caserta LC Frye EA Butt SL Laverack M Nooruzzaman M Covaleda LM. Spillover of highly pathogenic avian influenza H5n1 virus to dairy cattle. Nature. (2024) 634:669–76. doi: 10.1038/s41586-024-07849-4, PMID: 39053575 PMC11485258

[55] Kristensen C Larsen LE Trebbien R Jensen HE. The avian influenza a virus receptor Sa-Α2,3-gal is expressed in the porcine nasal mucosa sustaining the pig as a mixing vessel for new influenza viruses. Virus Res. (2024) 340:199304. doi: 10.1016/j.virusres.2023.199304, PMID: 38142890 PMC10793167

[56] Domańska-Blicharz K Świętoń E Świątalska A Monne I Fusaro A Tarasiuk K. Outbreak of highly pathogenic avian influenza a(H5n1) clade 2.3.4.4b virus in cats, Poland, June to July 2023. Euro Surveill. (2023) 28:2300366. doi: 10.2807/1560-7917.Es.2023.28.31.2300366 37535474 PMC10401911

[57] Wang J Yang J Ge J Hua R Liu R Li X. Newcastle disease virus-vectored West Nile fever vaccine is immunogenic in mammals and poultry. Virol J. (2016) 13:1–11. doi: 10.1186/s12985-016-0568-5, PMID: 27342050 PMC4920995

[58] Barnett KM Civitello DJ. Ecological and evolutionary challenges for wildlife vaccination. Trends Parasitol. (2020) 36:970–8. doi: 10.1016/j.pt.2020.08.006, PMID: 32952060 PMC7498468

[59] Edwards SJ Chatterjee HJ Santini JM. Anthroponosis and risk management: a time for ethical vaccination of wildlife? Lancet Microbe. (2021) 2:e230–1. doi: 10.1016/s2666-5247(21)00081-1, PMID: 33824953 PMC8016401

